# Support surfaces for intraoperative pressure injury prevention: systematic review with meta-analysis*

**DOI:** 10.1590/1518-8345.5279.3493

**Published:** 2021-11-08

**Authors:** Carolina Beatriz Cunha Prado, Elaine Alves Silva Machado, Karina Dal Sasso Mendes, Renata Cristina de Campos Pereira Silveira, Cristina Maria Galvão

**Affiliations:** 1 Universidade de Uberaba, Ciências da Saúde, Uberaba, MG, Brazil.; 2 Universidade de São Paulo, Escola de Enfermagem de Ribeirão Preto, PAHO/WHO Collaborating Centre for Nursing Research Development, Ribeirão Preto, SP, Brazil.; 3 Scholarship holder at the Conselho Nacional de Desenvolvimento Científico e Tecnológico/Ministério da Ciência, Tecnologia e Inovações, Brazil.

**Keywords:** Perioperative Nursing, Pressure Ulcer, Systematic Review, Meta-Analysis, Intraoperative Period, Equipment and Supplies, Enfermería Perioperatoria, Úlcera por Presión, Revisión Sistemática, Metaanálisis, Periodo Intraoperatorio, Equipos y Suministros, Enfermagem Perioperatória, Lesão por Pressão, Revisão Sistemática, Metanálise, Período Intraoperatório, Equipamentos e Provisões

## Abstract

**Objective:**

to evaluate evidence on effectiveness support surfaces for pressure injury prevention in the intraoperative period.

**Method:**

systematic review. The search for primary studies was conducted in seven databases. The sample consisted of 10 studies. The synthesis of the results was carried out descriptively and through meta-analysis.

**Results:**

when comparing low-tech support surfaces with regular care (standard surgical table mattress), the meta-analysis showed that there is no statistically significant difference between the investigated interventions (Relative Risk = 0.88; 95%CI: 0.30-2.39). The Higgins inconsistency test indicated considerable heterogeneity between studies (I^2^ = 83%). The assessment of the certainty of the evidence was very low. When comparing high-tech and low-tech support surfaces, the meta-analysis showed that there is a statistically significant difference between the interventions studied, with high-tech being the most effective (Relative Risk = 0.17; 95%CI: 0.05-0.53). Heterogeneity can be classified as not important (I^2^ = 0%). The assessment of certainty of evidence was moderate.

**Conclusion:**

the use of high-tech support surfaces is an effective measure to prevent pressure injuries in the intraoperative period.

## Abstract (es)

**Objetivo:**

evaluar las evidencias sobre superficies de apoyo efectivas para la prevención de lesión por presión durante el período intraoperatorio.

**Método:**

revisión sistemática. La búsqueda de estudios primarios se realizó en siete bases de datos. La muestra estuvo formada por 10 investigaciones. La síntesis de los resultados se realizó de forma descriptiva y mediante metaanálisis.

**Resultados:**

al comparar las superficies de apoyo de baja tecnología con la atención habitual (colchón de mesa quirúrgica estándar), el metaanálisis demostró que no hay diferencia estadísticamente significativa entre las intervenciones investigadas (Riesgo Relativo = 0,88; IC95%: 0,30-2,39). La prueba de inconsistencia de Higgins indicó una heterogeneidad considerable entre los estudios (I^2^= 83%). La valoración de la certeza de la evidencia fue muy baja. Al comparar las superficies de apoyo de alta y baja tecnología, el metaanálisis mostró que existe una diferencia estadísticamente significativa entre las intervenciones estudiadas, siendo las de alta tecnología las más efectivas (Riesgo Relativo = 0,17; IC95%: 0,05-0,53). La heterogeneidad se puede clasificar como no importante (I^2^ = 0%). La evaluación de la certeza de la evidencia fue moderada.

**Conclusión:**

el uso de una superficie de apoyo de alta tecnología es una medida eficaz para prevenir lesiones por presión en el período intraoperatorio.

## Abstract (pt)

**Objetivo:**

avaliar as evidências sobre superfícies de suporte efetivas para prevenção de lesão por pressão no período intraoperatório.

**Método:**

revisão sistemática. A busca dos estudos primários foi conduzida em sete bases de dados. A amostra foi composta por 10 pesquisas. A síntese dos resultados foi realizada na forma descritiva e por meio de metanálise.

**Resultados:**

na comparação de superfícies de suporte de baixa tecnologia com o cuidado usual (colchão padrão de mesa cirúrgica), a metanálise demonstrou que não existe diferença estatisticamente significante entre as intervenções investigadas (Risco Relativo = 0,88; IC95%: 0,30-2,39). O teste de inconsistência de Higgins indicou heterogeneidade considerável entre os estudos (I^2^ = 83%). A avaliação da certeza da evidência foi muito baixa. Na comparação de superfícies de suporte de alta tecnologia com as de baixa tecnologia, a metanálise evidenciou que existe diferença estatisticamente significante entre as intervenções estudadas, sendo as de alta tecnologia as mais efetivas (Risco Relativo = 0,17; IC95%: 0,05-0,53). A heterogeneidade pode ser classificada como não importante (I^2^ = 0%). A avaliação da certeza da evidência foi moderada.

**Conclusão:**

o uso de superfície de suporte de alta tecnologia é a medida efetiva para prevenção de lesão por pressão no período intraoperatório.

## Introduction

Pressure injury (PI) is an adverse event that can affect the surgical patient. In recently published clinical guidelines, information based on research results indicated that the incidence of this type of injury, directly attributable to the surgical anesthetic procedure, can range from 4% to 45%^(1)^. This variability of data must be interpreted with caution, since in the intraoperative period skin changes due to the appearance of PI may take a while to manifest, several hours or even three to five days after surgery. This condition can generate an underestimated number of this type of injury resulting from the surgical anesthetic procedure; in addition, it is commonly attributed to the postoperative period or confused with burns^(1)^.

In the intraoperative period, the appearance of PI is related to different factors, which can be classified as intrinsic to the patient (for example, age, Body Mass Index and presence of chronic disease), extrinsic (for example, exposure to pressure, especially in bone prominences, friction, shear and altered microclimate) and related to the surgical anesthetic procedure (duration of the surgical anesthetic procedure, type of surgical position, among others)^(2-4)^.

In the literature there is evidence of the importance of using support surfaces for the prevention of PI in the intraoperative period. These devices can be mattresses, overlays or specific pads for different parts of the human body, and they can be made of foam, gel, viscoelastic polymer, air or fluids^(1,5-6)^. Support surfaces can be classified into high tech and low tech. The first one is dynamic, capable of changing the pressure distribution with or without load applied and powered by an energy source (for example: alternating pressure overlay). On the other hand, the low-tech surface is not powered by electricity and adapts to the shape of the body, distributing body weight over a large area (for example: dry viscoelastic overlay)^(7)^. On the other hand, there are knowledge gaps, which are the most effective support surfaces for use in the operating room^(7-8)^.

The perioperative nurse has a fundamental role in the assessment of the patient before the surgical anesthetic procedure and in the identification of predisposing factors for the occurrence of skin lesions, including PI. In the intraoperative period, the planning and implementation of care for the prevention of PI are crucial for the reduction of complications associated with this type of injury, such as: intense pain in the postoperative period, not related to the surgical site; patient dissatisfaction; the extension of the length of stay; the increase in the expenses of the public/private health system^(9)^.

This systematic review was conducted in an attempt to contribute to the advancement of knowledge about the problem in question. In addition to providing support for nurses’ decision-making in clinical practice, with a view to increasing the quality of care provided and reducing costs, mainly related to the treatment of PI and the use of appropriate technology in the operating room. Thus, the delimited objective was to evaluate the evidence on effectiveness support surfaces for the prevention of pressure injuries in the intraoperative period.

## Method

### Type of study

This is a systematic review of health interventions and was conducted based on the recommendations of the Cochrane Collaboration. The following steps were taken: 1) elaboration and registration of the review protocol; 2) delimitation of the review question; 3) definition of eligibility criteria; 4) search and selection of studies; 5) data collection; 6) synthesis and presentation of the results of the systematic review^(10)^. The Preferred Reporting Items for Systematic Review and Meta-Analyses (PRISMA) checklist guidelines were also adopted to report the systematic review^(11)^.

The review protocol was registered in the International Prospective Register of Systematic Reviews (PROSPERO). The registration number is CRD42019131271 and the protocol can be accessed at the website (https://www.crd.york.ac.uk/prospero/display_record.php)

### Setting

The systematic review was conducted in the city of Ribeirão Preto, state of São Paulo, Brazil.

### Period

The systematic review took place from January to November 2020.

### Population

The delimited review question was: “what are the effective support surfaces for the prevention of pressure injuries in patients during the intraoperative period?”. The question followed the components of the acronym PICOT (population, intervention, comparison, outcome and time), being P = surgical patient; I = tested support surface; C = standard care (non-use of support surface) or support surface different from the one tested; O = pressure injury prevention; T = intraoperative period.

### Selection criteria

In the systematic review, primary studies that met the components of the PICOT strategy were included, and those in which the population consisted of patients under 18 years old or volunteers were excluded. Systematic reviews of the effectiveness of health interventions advocated by the Cochrane Collaboration traditionally focus on the inclusion of randomized controlled trials. However, this organization also discusses the inclusion, in this type of review, of non-randomized studies of interventions^(10)^. Given the above and the diversity of non-randomized study designs, the reviewers delimited the inclusion of randomized controlled trials and non-randomized studies, whose authors investigated the effectiveness of support surfaces in preventing pressure injuries in the intraoperative period. With regard to non-randomized studies, studies that in the design presented at least two comparative groups (for example, a control group and an intervention group) were selected. It is also noteworthy that for the selection of primary studies, limitations of language or period of publication were not established.

### Sample definition

The databases selected for the search of primary studies were PubMed, Cumulative Index to Nursing and Allied Health Literature (CINAHL), Cochrane Central Register of Controlled Trials (CENTRAL), EMBASE, Scopus, Web of Science, and Latin American and Latin American Literature Caribbean in Health Sciences (LILACS).

Before performing the final searches of the primary studies in the selected databases, several combinations were performed using the controlled descriptors, keywords and the Boolean operators AND and OR, this was done in order to identify the largest possible number of publications. For this step, the combinations adopted the five components of the PICOT strategy. However, it was observed that the removal of P and C elements allowed the increase of the search amplitude. Thus, the combination I AND O AND T was used, and in four databases, PubMed, CENTRAL, Web of Science and Scopus, the controlled descriptors were delimited from the Medical Subject Headings (MeSH) and the search strategies adopted were: I - “Equipment and Supplies”[Mesh] OR “Supplies and Equipment” OR “Apparatus and Instruments” OR “Instruments and Apparatus” OR “Supplies” OR “Inventories” OR “Inventory” OR “Medical Devices” OR “Medical Device” OR “Device, Medical” OR “Devices, Medical” OR “Devices” OR “Device” OR “Equipment” OR “support surface” OR “foam mattress” OR “gel mattress” OR “visco-elastic polyether foam mattress” OR “visco-elastic polyurethane mattress” OR “polymers” OR “mattress” OR “foam” OR “viscoelastic” OR “pillows polyurethane foam” OR “rubber foam” OR “pillows” OR “cushion” OR “overlay” OR “pad” OR “Dry viscoelastic Polymer”; O - “Pressure Ulcer”[Mesh] OR “Pressure Ulcers” OR “Ulcer, Pressure” OR “Ulcers, Pressure” OR “Bedsore” OR “Bedsores” OR “Pressure Sore” OR “Pressure Sores” OR “Sore, Pressure” OR “Sores, Pressure” OR “Bed Sores” OR “Bed Sore” OR “Sore, Bed” OR “Sores, Bed” OR “Decubitus Ulcer” OR “Decubitus Ulcers” OR “Ulcer, Decubitus” OR “Ulcers, Decubitus” OR “Interface pressure” OR “Pressure ulcer Prevention and control” OR “intraoperative pressure injuries” OR “intraoperatively acquired pressure ulcer” OR “Wounds and Injuries”[Mesh] and T - “Intraoperative Period”[Mesh] OR “Intraoperative Periods” OR “Period, Intraoperative” OR “Periods, Intraoperative”. In the other databases, CINAHL, EMBASE and LILACS, the search strategies used were similar, however the controlled descriptors used were in accordance with the base vocabulary, namely: CINAHL Headings, Emtree and Descriptors in Health Sciences (DeCS).

At the end of the search for primary studies in all selected databases, the results were exported to EndNote Basic (desktop version) for the removal of duplicates^(12)^. Then, all citations from the reference manager were imported into the Rayyan technology platform of the Qatar Computing Research Institute (QCRI), specifically aimed at the study selection phase among reviewers. Thus, allowing the blinding between these and the monitoring of the selection process by the main researcher. This platform can be accessed through an electronic address (https://rayyan.qcri.org/welcome) or as an application for smartphones^(13)^.

Titles and abstracts of primary studies identified in the databases and imported from EndNote Basic to the Rayyan platform were independently assessed by two reviewers to determine which studies met the aforementioned eligibility criteria. The reading of the primary studies, in full, was also carried out independently by two reviewers. In those cases where there was disagreement between reviewers, a third reviewer was consulted to solve the question.

The search and selection of primary studies that were included in the review sample took place from February to April 2020. Through a manual search, the main reviewer tried to identify, in the reference list of each study included in the review, other studies that could answer the guiding question. However, no study was selected.

### Data collection

A standard form was developed to collect data from the studies included in the systematic review. The script items were: authors; study title; year of publication; journal name; goal; sample; inclusion and exclusion criteria for the investigated population; randomization; blinding; type of anesthesia and duration; type of surgery and duration; intervention/experimental group; group control; number of patients who had pressure injury at the end of the study; statistical analysis; main results; conclusion. Data collection was again carried out by two reviewers, independently, in May and June 2020. To solve items and/or information that presented divergences, meetings were scheduled between reviewers for discussion and resolution of divergent aspects until consensus.

### Data analysis

To analyze the risk of bias of the randomized controlled trials included in the review (n=6), the free tool named Revised Cochrane risk-of-bias tool for randomized trials (RoB 2), which is proposed by the Cochrane Collaboration^(10)^ was adopted. This tool has five domains, namely: bias resulting from the randomization process; bias due to deviations from intended interventions; bias from missing outcome data; bias from the measurement of the outcome; bias from the selection of the reported result. Such analysis was performed by two reviewers, independently. Through meetings, the results of each evaluated study and the doubts were discussed until the reviewers reached consensus.

To assess the methodological quality of the non-randomized studies (n=4), the quasi-experimental study tool proposed by the Joanna Briggs Institute (JBI) was used. The tool is called JBI Critical Appraisal Checklist for Quasi-Experimental Studies, and is composed of nine questions. For each question, the reviewer answers yes, no, unclear or not applicable. The questions are aimed at assessing the study’s internal validity and risk of bias (selection of participants, conduction and analysis of results)^(14)^. In this analysis, two reviewers also independently assessed the four studies. Then, a meeting was held to discuss doubts and final evaluation of the research. The adopted tool does not have a scoring system for the general evaluation of the study.

The summary of the review results was carried out in descriptive form and through meta-analysis. To perform the meta-analysis, randomized controlled trials were grouped according to the support surfaces investigated by the researchers. The delimited meta-analysis analysis model was the random effect, using the software Review Manager (RevMan) version 5.3 of the Cochrane Collaboration.

The assessment of the certainty of the evidence was performed using the Grading of Recommendations Assessment, Development and Evaluation (GRADE). This assessment is performed for each outcome analyzed. In this review, the outcome is the development of pressure injury related to the use of support surfaces using the evidence available in the literature. The certainty of the evidence can be assessed as high (strong confidence that the true effect is close to that estimated), moderate (moderate confidence in the estimated effect), low (limited confidence in the effect estimate) and very low (very limited confidence in the estimate of the effect)^(15)^. The assessment of the certainty of the evidence was performed using the GRADEpro software (https://www.gradepro.org)

## Results

**Figure 1:**
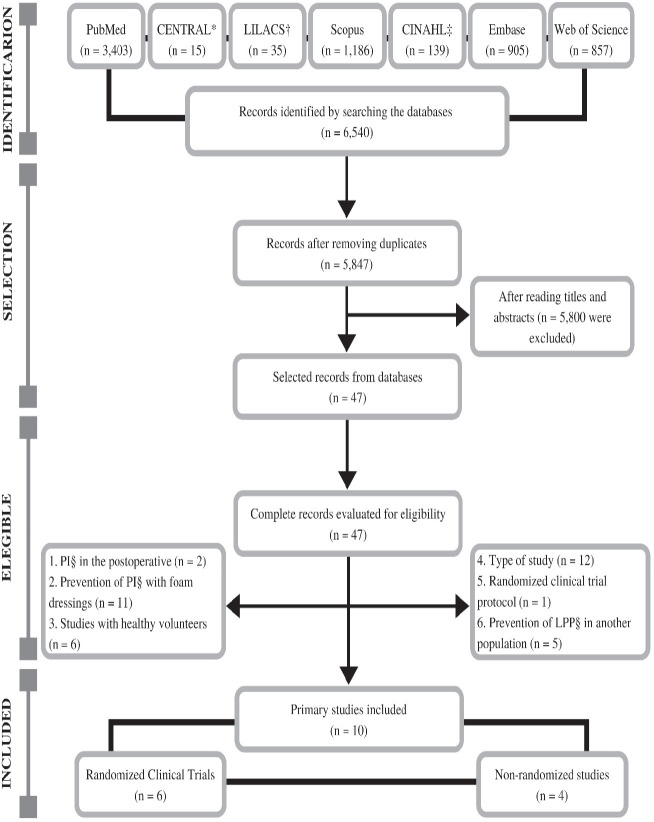
Flowchart of the selection process of primary studies included in the systematic review adapted from Preferred Reporting Items for Systematic Review and Meta-Analyses (PRISMA). Ribeirão Preto, SP, Brazil, 2020 *CENTRAL = Cochrane Central Register of Controlled Trials;^†^LILACS = Latin American and Caribbean Literature in Health Sciences;^‡^CINAHL = Cumulative Index to Nursing and Allied Health Literature;^§^PI = Pressure injury

Source: Moher, et al.^(11)^

In Figure 1, the detailed flowchart of the selection process of the primary studies included in the systematic review is presented. Thus, the review sample consisted of 10 studies, with six randomized controlled trials and four non-randomized studies.

**Figure 2:**
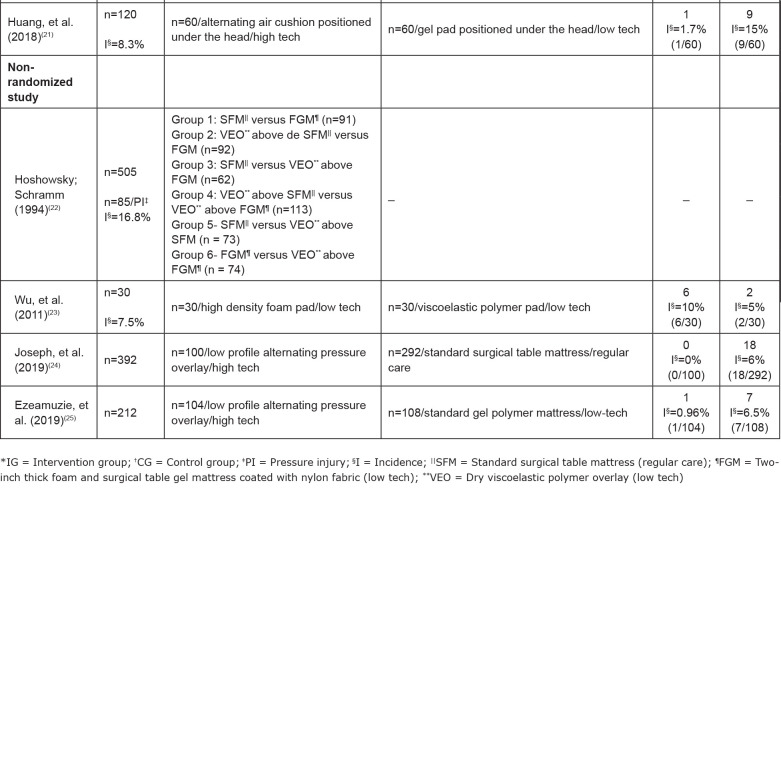
Characterization of primary studies included in the systematic review. Ribeirão Preto, SP, Brazil, 2020

In Figure 2, the descriptive synthesis of the primary studies was presented. The following data were indicated: authors and year of publication of the research; sample; support surfaces tested in the intervention and control groups; number of PI in each group; the incidences of the analyzed outcome. Missing data were not described by the authors of the included studies.

**Figure 3:**
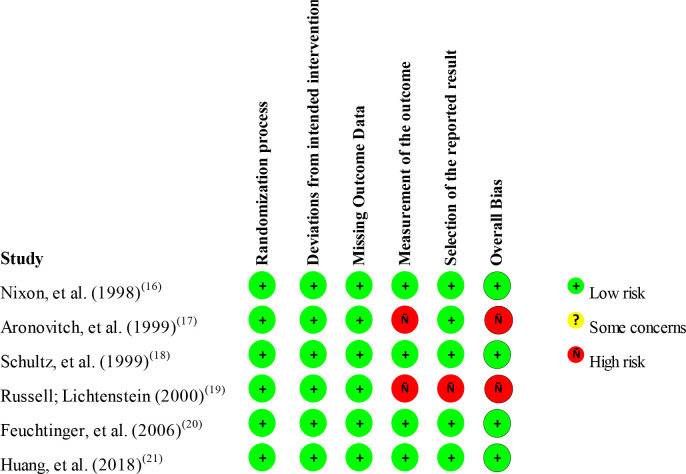
Risk of bias assessment of randomized controlled trials in each domain of the Revised Cochrane risk-of-bias tool for randomized trials (RoB 2). Ribeirão Preto, SP, Brazil, 2020

Figure 3 shows the result of the risk of bias assessment using the RoB 2 tool, which was presented for each of the six randomized controlled trials included in the systematic review.

Of the six randomized controlled trials, 66.7% (n=4) were considered to be at low risk of bias and 33.3% (n=2) were considered to be at high risk of bias. In two studies^(17,19)^ the bias domain in the measurement of results was evaluated as being of high risk, since there was no information about blinding of the result evaluators, that is, the evaluator could know which was the participant’s group and perform less rigorous evaluation for patients in the experimental group regarding the outcome, in this case, the development of PI. In one study^(19)^ the bias domain in the selection of reported outcome was also assessed as high risk, that is, researchers reported outcome measures selectively favorable to the intervention of the experimental group.

The assessment of the methodological quality of the non-randomized studies (n=4) was performed using the JBI Critical Appraisal Checklist for Quasi-Experimental Studies, as already mentioned, this tool does not have a scoring system. Thus, of the nine questions that make up the checklist, in two studies^(24-25)^, eight questions received the answer “yes” in the assessment carried out by the reviewers; in one study^(22)^, seven questions received “yes”; and in the other research^(23)^, five questions received “yes”, and in the evaluation, three questions received the answer “not applicable”, since the questions were related to follow-up and comparison between the control and experimental groups. In this study, the support surfaces were tested on the same patient, and the high-density foam pad was tested under the right chest and the right iliac crest (experimental intervention), and the viscoelastic polymer pad was tested under the left chest and the left iliac crest (control intervention).

**Figure 4:**
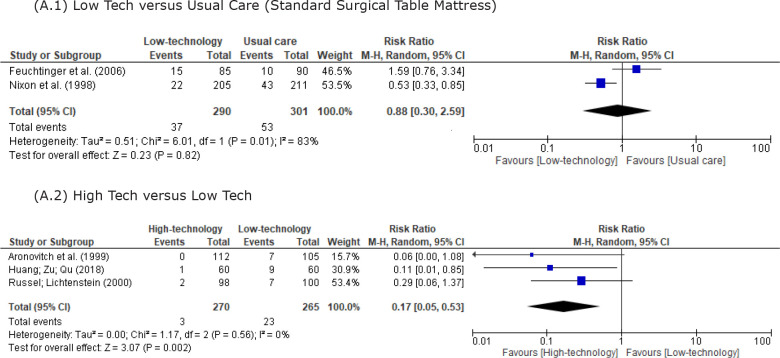
Forest plots from meta-analyses addressing pressure injury prevention interventions. Ribeirão Preto, SP, Brazil, 2020

In the meta-analysis, only randomized controlled trials with similar characteristics regarding the surfaces tested in the intervention and control groups were included. As already mentioned, the outcome considered to assess the effectiveness of the support surfaces was the development of pressure injury in the intervention and control groups. In Figure 4, two meta-analyses were presented. The first considers clinical trials in which the authors tested low-tech support surfaces in comparison with usual care (standard surgical table mattress) (Figure 4 A.1). In the second meta-analysis, the clinical trials in which researchers investigated high-tech support surfaces compared to low-tech support surfaces are considered (Figure 4 A.2). The Relative Risk (RR) was indicated in the last column of the forest plots.

In Figure 4 A.1, when comparing low-tech support surfaces with usual care (standard surgical table mattress), the interpretation of the meta-analysis indicates that there is no statistically significant difference between the investigated interventions (RR = 0, 88; 95%CI: 0.30-2.39). On the other hand, in Figure 4 A.2, when comparing high-tech and low-tech support surfaces, the interpretation of the meta-analysis shows that there is a statistically significant difference between the investigated interventions, with the high-tech ones being the most effective (RR = 0.17; 95%CI: 0.05-0.53).

In Figure 4 A.1, the Higgins inconsistency statistical test (I^2^) indicated considerable heterogeneity between studies (I^2^ = 83%). On the other hand, on Figure 4 (A.2), heterogeneity can be classified as unimportant (I^2^ = 0%).

In Table 1, the assessment of certainty of evidence by the GRADE system was presented. As explained above, this assessment is performed for each outcome, in the case of this review, the development of pressure injury. Thus, when comparing low-tech support surfaces with usual care, the certainty of the evidence was very low (very limited confidence in the estimation of the effect), as it presented very serious inconsistency, that is, considerable heterogeneity (I^2^ = 83%). Furthermore, the imprecision was also rated as very severe due to variation in the effect estimate. When comparing high-tech and low-tech support surfaces, the certainty of the evidence was moderate (moderate confidence in the estimated effect), since two randomized controlled trials were evaluated at high risk of bias.

**Table 1:** Synthesis of the assessment of the certainty of evidence, according to the Grading of Recommendations Assessment, Development and Evaluation (GRADE). Ribeirão Preto, SP, Brazil, 2020

Certainty of evidence							Number of patients		Effect		
Number of study	Type of study	Risk of bias	Inconsistency	Indirect evidence	Imprecision	Other considerations	**I** *	**C** ^†^	Relative (95% CI^‡^)	Absolute (95% CI^‡^)	Certainty
Incidence of Pressure Injury/Low Technology versus Standard Surgical Table Mattress											
2	RCT^§^	not serious	very serious^||^	not serious	very serious^¶|^	none	37/290 (12.8%)	53/301 (17.6%)	not estimable	20 plus *per* 1,000 (from 140 minus to 180 plus)	⊕⃝⃝⃝ Very low
Incidence of Pressure Injury/High Tech versus Low Tech											
3	RCT^§^	serious**	not serious	not serious	not serious	none	3/270 (1.1%)	23/265 (8.7%)	RR^††^ = 0.17 (0.05 to 0.53)	72 minus *per* 1,000 (from 82 minus to 41 minus)	⊕⊕⊕⃝ Moderate

* I = Intervention;

† C = Control;

‡ CI = Confidence interval;

§ RCT = Randomized controlled trial;

|| The justification for the assessment is that the Higgins inconsistency test (I2=83%) indicated considerable heterogeneity between studies;

¶| The justification for the assessment is that the effect estimate varies greatly;

** The justification for the assessment is that two randomized controlled trials were considered to be at high risk of bias;

†† RR = Relative risk

## Discussion

To make the discussion of the evidenced results easier, three categories were defined (the first one comparing low-tech support surfaces with regular care, that is, standard surgical table mattress), in addition, two randomized controlled trials were grouped^(16,20)^. In a study^(20)^ the results led to the interruption of the research, since the patients in the intervention group (overlay of thermoactive viscoelastic foam of 4 cm) had a higher number of PI, although the difference between the groups was not statistically significant. In another study^(16)^, the results showed that the use of a dry viscoelastic polymer pad was more effective in preventing PI compared to regular care (OR=0.46; 95%CI: 0.26-0.82; p=0.01).

In a quasi-experimental study carried out in Brazil, the authors evaluated the interface pressure of support surfaces in bony prominences, at specific points (occipital, subscapular, sacral and calcaneal regions) in 20 healthy volunteers in supine position on a surgical table. Seven different combinations were evaluated, namely: standard surgical table mattress without overlaying; the viscoelastic polymer overlay; three overlays of 5 cm thick sealed foam at densities 28, 33 and 45 kg/m^3^; two overlays of soft foam 5 cm thick and densities 28 and 18 kg/m^3^. The mean interface pressure of the viscoelastic polymer overlay was higher compared to the other surfaces tested, including the standard surgical table mattress (p<0.001)^(5)^.

The second category (high-tech support surfaces versus low-tech surfaces) included three randomized controlled trials^(17,19,21)^ and two non-randomized studies^(24-25)^. In all studies, the high-tech surfaces tested were alternating pressure devices from different manufacturers. In two randomized controlled trials^(17,19)^, the MicroPulse^®^ System alternating air overlay (MicroPulse, Inc., Portage, Michigan, USA) was tested. In non-randomized studies^(24-25)^, low-profile alternating pressure overlap was investigated (Dabir Micropressure Operating Table Surface^®^, Dabir Surfaces, Chicago, Illinois, USA). In a randomized controlled trial^(21)^, the surface tested was an alternating air cushion from the Chinese manufacturer WeXuan Co.

In four studies, the results showed the superiority of a high-tech support surface in relation to low-tech surfaces in the prevention of PI in the intraoperative period^(17,21,24-25)^. In a randomized controlled trial^(19)^, the experimental group (high-tech support surface) had a lower incidence of PI (2/98) than the control group (7/100), however, there was no statistically significant difference between the groups (p=0.172).

In conducting the two non-randomized studies included in the review, there are similarities in terms of research design, population and tested support surfaces^(24-25)^. In both, in the experimental group, low-profile alternating pressure overlay was tested. This overlay incorporates hundreds of supporting nodules arranged in rows that periodically inflate with air, so the patient’s weight is distributed over small nodal points of alternating contact. Alternate rows are interconnected so that the overlay has two areas that are alternately inflated. Inflation/deflation of the rows is computer controlled and provides temporary localized relief of micropressure in areas of the body lying above deflated nodules. The overlay was placed on top of the standard operating table mattress, before starting the surgery.

The operating room is considered as a place of risk for the development of PI, due to strict restrictions specific to the environment, namely: the inability to reposition the patient during the anesthetic surgical procedure for pressure relief and the need of permanence on a stable support surface, generally implying the use of a relatively rigid padding material, resulting in the exposure of the body to tissue deformation conditions. In this context, low-profile alternating pressure overlay was designed for use in surgery, which brought technological advances in a field in which contemporary technology is generally poor^(26)^.

In the last category (comparison between low-tech support surfaces) two non-randomized studies^(22-23)^ and one randomized controlled trial were included^(18)^. In a non-randomized study^(22)^, two operating table mattresses and an overlay of dry viscoelastic combined in different ways were tested with the participation of 505 patients (divided into six groups). Regarding PI development, dry viscoelastic polymer overlay was more effective than foam and gel or standard mattresses.

In the other non-randomized study^(23)^, two support surfaces were tested on the same patient, and on the right side a high-density foam pad (32 kg/m^3^), 50% resilience and 10 cm thickness was applied (chest and iliac crest) and on the left side the viscoelastic polymer pillow (Action^®^, model 40700; Action, Hagerstown, Maryland, USA), two-cm-thick, also on the chest and iliac crest. Mean pressures and peak pressures were significantly lower at the points evaluated with the viscoelastic polymer pad, compared to the points tested with the high-density foam pad. However, the results did not show a statistically significant difference in the incidence of pressure injury between the two support surfaces tested (OR=0.47, 95% CI, 0.11-1.99).

In the randomized controlled trial^(18)^, also included in this category, patients in the control group used devices according to the criteria of each nurse. Options included gel pads, egg box foam mattress and “foam donuts” for heels and elbows. The patients in the intervention group were placed on a special foam cover with a 25% indentation force (IF) of 30 pounds and a density of 1.3 (specification considered ideal). The number of participants in the experimental group (55/206) showed significantly higher occurrence of PI than those in the control group (34/207) (p=0.0111), indicating that the special foam surface that was tested was not effective in preventing this type of injury.

The standard surgical table mattress is usually made of two-inch (5.08 cm) elastic foam and covered with black vinyl fabric. Despite its excellent stability, there is evidence that this type of surface contributes to the development of PI. On the other hand, mattresses made with high-specification foam can reduce the development of this type of injury. Thus, the multi-layer smooth surfaces allow the patient to sink into the underlayer and wrap around the body to increase the contact area by up to 60%. Such properties help to distribute pressure over a larger area. Bi-elastic layers also reduce skin creases and shear forces^(27)^.

The support surface must have the best characteristics to provide effective pressure redistribution, which are: lowest mean interface pressure, lowest peak interface pressure and highest skin contact area. Based on these assumptions, researchers conducted a comparative descriptive study with volunteers to investigate four types of support surfaces, with the aim of identifying the most effective surface for pressure redistribution in prolonged surgical procedures. The surfaces tested were: a) standard surgical table surface, made of three-layer viscoelastic foam; b) static air-inflated seat cushion that was used under the sacral area and placed on the standard surgical table surface; c) two-layer surgical table surface, with the upper layer of gel and the lower layer of high-density foam; d) surgical surface for simulating fluid immersion. The results indicated that, although all surfaces had similar mean interface pressures, the air-inflated static seat cushion had the best pressure redistribution properties in the sacral region, compared to the other surfaces tested^(28)^.

The results of the systematic review showed that high-tech support surfaces are more effective than low-tech ones (evidence through meta-analysis) in the intraoperative period. These results have implications for clinical practice, since the implementation of this technology requires a high financial investment from the health service, that is, a reality that is probably distant in developing countries. On the other hand, when comparing low-tech support surfaces with regular care, the assessment of the certainty of the evidence was very low, indicating that conducting further research is likely to change the estimate of the effect. In short, conducting well-designed randomized controlled trials, testing low-tech support surfaces, may contribute to decision-making by perioperative nurses in clinical practice, especially in developing countries. The evidence generated may help this professional in planning and implementing effective support surfaces for the prevention of PI in the intraoperative period.

Despite the extensive search carried out in seven databases, as well as the absence of time and language limitations, the identification of a small number of randomized controlled trials can be considered a limitation, since this type of study is the most suitable for investigating the effectiveness of health interventions. In addition to this aspect, the researchers delimited the inclusion of primary studies indexed in the selected databases, that is, the non-inclusion of gray literature; this was due to the difficulty of accessing and handling this type of material. This decision can also be considered as a limitation.

## Conclusion

The results of the meta-analysis conducted indicated that when comparing low-tech support surfaces with regular care, there was no statistically significant difference. Furthermore, the considerable heterogeneity between the studies and the very low certainty of the evidence is highlighted, indicating that the conduct of other researches is likely to change the estimate of the effect.

When comparing high-tech and low-tech support surfaces, there was a statistically significant difference between the investigated interventions, with high-tech being the most effective. Furthermore, it is noteworthy that heterogeneity can be classified as not important and the assessment of the certainty of the evidence was moderate.

Based on the above, it is recommended to conduct well-designed randomized controlled trials to investigate support surfaces for the prevention of pressure injuries in the intraoperative period, considering the cost-effectiveness of the technology.

## References

[1] Emily Haesler, editor (2019). Prevention and treatment of pressure ulcers/injuries: clinical practice
guideline [Internet].

[2] Engels D, Austin M, McNichol L, Fencl J, Gupta S, Kazi H (2016). Pressure ulcers: factors contributing to their development in the
OR. AORN J.

[3] Xiong C, Gao X, Ma Q, Yang Y, Wang Z, Yu W (2019). Risk factors of intraoperative pressure injuries in patients
undergoing digestive surgery: a retrospective study. J Clin Nurs.

[4] Yoshimura M, Ohura N, Santamaria N, Watanabe Y, Akizuki T, Gefen A (2020). High body mass index is a strong predictor of intraoperative
acquired pressure injury in spinal surgery patients when prophylactic film
dressings are applied: a retrospective analysis prior to the BOSS
trial. Int Wound J.

[5] Oliveira KF, Pires OS, De-Mattia AL, Barichello E, Galvão CM, Araújo CA (2018). Influence of support surfaces on the distribution of body
interface pressure in surgical positioning. Rev. Latino-Am. Enfermagem.

[6] Karg P, Ranganathan VK, Churilla M, Brienza D (2019). Sacral skin blood flow response to alternating pressure operating
room overlay. J Tissue Viability.

[7] McInnes E, Jammali-Blasi A, Bell-Syer SE, Dumville JC, Middleton V, Cullum N (2015). Support surfaces for pressure ulcer prevention. Cochrane Database Syst Rev.

[8] Oliveira KF, Nascimento KG, Nicolussi AC, Chavaglia SRR, Araújo CA, Barbosa MH (2017). Support surfaces in the prevention of pressure ulcers in surgical
patients: an integrative review. Int J Nurs Pract.

[9] Khong BPC, Goh BC, Phang LY, David T (2020). Operating room nurses’ self-reported knowledge and attitude on
perioperative pressure injury. Int Wound J.

[10] Higgins JPT, Thomas J (2019). Cochrane handbook for systematic reviews of interventions.

[11] Moher D, Liberati A, Tetzlaff J, Altman DG, PRISMA Group (2009). Preferred reporting items for systematic reviews and
meta-analyses: the PRISMA statement. PLoS Med.

[12] Mendes KDS, Silveira RCCP, Galvão CM (2019). Uso de gerenciador de referências bibliográficas na seleção dos
estudos primários em revisão integrativa. Texto Contexto Enferm.

[13] Ouzzani M, Hammady H, Fedorowicz Z, Elmagarmid A (2016). Rayyan - a web and mobile app for systematic
reviews. Syst Rev.

[14] Tufanaru C, Munn Z, Aromataris E, Campbell J, Hopp L, Aromataris E, Munn Z (2020). JBI Manual for evidence synthesis.

[15] Ministério da Saúde (BR) (2014). Diretrizes metodológicas: Sistema GRADE - manual de graduação da
qualidade da evidência e força de recomendação para tomada de decisão em
saúde.

[16] Nixon J, McElvenny D, Mason S, Brown J, Bond S (1998). A sequential randomised controlled trial comparing a dry
visco-elastic polymer pad and standard operating table mattress in the
prevention of postoperative pressure sores. Int J Nurs Stud.

[17] Aronovitch SA, Wilber M, Slezak S, Martin T, Utter D (1999). A comparative study of an alternating air mattress for the
prevention of pressure ulcers in surgical patients. Ostomy Wound Manage.

[18] Schultz A, Bien M, Dumond K, Brown K, Myers A (1999). Etiology and incidence of pressure ulcers in surgical
patients. AORN J.

[19] Russell JA, Lichtenstein SL (2000). Randomized controlled trial to determine the safety and efficacy
of a multi-cell pulsating dynamic mattress system in the prevention of
pressure ulcers in patients undergoing cardiovascular
surgery. Ostomy Wound Manage.

[20] Feuchtingern J, Bie R, Dassen T, Halfens R (2006). A 4-cm thermoactive viscoelastic foam pad on the operating room
table to prevent pressure ulcer during cardiac surgery. J Clin Nurs.

[21] Huang W, Zhu Y, Qu H (2018). Use of an alternating inflatable head pad in patients undergoing
open heart surgery. Med Sci Monit.

[22] Hoshowsky VM, Schramm CA (1994). Intraoperative pressure sore prevention: an analysis of bedding
materials. Res Nurs Health.

[23] Wu T, Wang ST, Lin PC, Liu CL, Chao YFC (2011). Effects of using a high-density foam pad versus a viscoelastic
polymer pad on the incidence of pressure ulcer development during spinal
surgery. Biol Res Nurs.

[24] Joseph J, McLaughlin D, Darian V, Hayes L, Siddiqui A (2019). Alternating pressure overlay for prevention of intraoperative
pressure injury. J Wound Ostomy Continence Nurs.

[25] Ezeamuzie O, Darian V, Katiyar U, Siddiqui A (2019). Intraoperative use of low-profile alternating pressure mattress
for prevention of hospital acquired pressure injury. Perioper Care Oper Room Manag.

[26] Gefen A (2020). Minimising the risk for pressure ulcers in the operating room
using a specialised low-profile alternating pressure overlay. Wounds Int [Internet].

[27] Scott SM (2016). Perioperative pressure injuries: protocols and evidence-based
programs for reducing risk [Internet]. https://www.psqh.com/analysis/perioperative-pressure-injuries-protocols-and-evidence-based-programs-for-reducing-risk/.

[28] Kirkland-Walsh H, Teleten O, Wilson M, Raingruber B (2015). Pressure mapping comparison of four OR surfaces. AORN J.

